# Teacher Perceptions of Their Curricular and Pedagogical Shifts: Outcomes of a Project-Based Model of Teacher Professional Development in the Next Generation Science Standards

**DOI:** 10.3389/fpsyg.2017.00989

**Published:** 2017-06-16

**Authors:** David J. Shernoff, Suparna Sinha, Denise M. Bressler, Dawna Schultz

**Affiliations:** ^1^Center for Mathematics, Science, and Computer Education Piscataway, Rutgers University, PiscatawayNJ, United States; ^2^Department of School Psychology, Graduate School of Applied and Professional Psychology, Rutgers University, PiscatawayNJ, United States

**Keywords:** NGSS, PBL, teacher professional development, curriculum, pedagogy, science education, standards

## Abstract

In this study, we conducted a model of teacher professional development (PD) on the alignment of middle and high school curricula and instruction to the Next Generation Science Standards (NGSSs), and evaluated the impact of the PD on teacher participants’ development. The PD model included a 4-day summer academy emphasizing project-based learning (PBL) in the designing of NGSS-aligned curricula and instruction, as well as monthly follow-up Professional Learning Community meetings throughout the year providing numerous opportunities for teachers to develop and implement lesson plans, share results of lesson writing and implementation (successes and challenges), provide mutual feedback, and refine curricula and assessments. Following the summer academy, six female teachers were interviewed about their current conceptualizations of NGSS, the extent of curricular shifts made that are required by NGSS, their self-perceptions regarding their level of accomplishment in curriculum writing, and the benefits of the PD in reaching their goals related to NGSS. Interviews were supplemented with an analysis of lesson plans written while participating in the PD program. The interviewed teachers suggested that they had made important conceptual and pedagogical shifts required by NGSS as they participated in the PD, and also noted a variety of challenges as they made this shift. While all teachers were relative novices at NGSS curriculum writing before the PD, most of the teachers interviewed felt that they had achieved the status of an “accomplished novice” following the summer academy. An analysis of their written lessons suggested a great range in the extent to which teachers effectively applied their understanding of NGSS to write lessons aligned to NGSS. Interviewed teachers believed that the PD model was helpful to their development as science teachers, and all reported that there were no aspects of the PD that were not helpful. Even though most teachers obtained a basic understanding and conceptualization of NGSS and PBL, their application of this understanding in their curriculum writing varied. The present study may help to inform future efforts to support teachers to align curricula and instruction to NGSS through teacher PD.

## Introduction

The Next Generation Science Standards (NGSSs) calls for a new approach to science education, helping students to act, think, and reason like scientists and engineers, i.e., make hypotheses, gather data, experiment, analyze data, draw conclusions from evidence, form arguments based on findings, and design solutions. This active, discovery-based approach is widely embraced by the educational and research community. However, it poses a daunting challenge: as school districts are expected to align instruction and curricula to NGSS and teachers are held accountable for this, there is a dire need for professional development (PD) to help teachers obtain this goal. PD is needed to help teachers to design high quality curricula and generate instruction aligned to NGSS. However, both good models of curricular units aligned to NGSS and good models of PD designed to assist teachers in creating them are in short supply. Designing and sharing these models has become a matter of some urgency. In the region where this study was conducted, high and middle schools were expected to be compliant with NGSS requirements by fall 2016, with elementary schools needing to comply by fall, 2017.

The primary goal of the present research project was to design, implement, and evaluate a PD model to address the major shifts in science education as curriculum and assessments are rewritten to align with the NGSS. This model sought to leverage project-based learning (PBL) specifically, and constructivist, learner-centered educational approaches more generally, as supported by research and theory in educational psychology (e.g., Piaget, 1968; Bereiter and Scardamalia, 1987; Brown et al., 1989; Collins et al., 1991; Blumenfeld et al., 1991; Scardamalia and Bereiter, 1991, 1994; Ames, 1992; McCombs, 1993, 1997; American Psychological Association [APA], 1997; Schunk, 2008).

The project team consisted of the Principal Investigator (PI, first author), two research associates, and PD specialist from a STEM education and research center at a large university on the East Coast of the U.S.; it also included a science coordinator from a nearby partnering school district. The project team prepared the teacher PD program, which included a 4-day summer academy and follow-up Professional Learning Community meetings (PLCs), to meet the needs of the partnering district. The PD was designed to focus on unpacking the NGSS standards and mapping them to curricular units, and strategies for curricular and instructional alignment with the NGSS. The summer academy provided an overview of project-based pedagogical practices and engineering design challenges, and the opportunity for collaborative writing of new 6th–12th grade science curricula starting in fall 2015. The PLCs were designed for continued reflection, sharing, and feedback of their newly developed NGSS-aligned units.

In addition to reporting on the pertinent details of our NGSS PD model, we report on evaluative research regarding the effectiveness of the PD model and areas of improvement. More specifically, our research questions were:

(1) How did teachers develop throughout the course of the PD project in terms of understanding the conceptual and pedagogical shifts inherent to NGSS? What were their greatest challenges?
(2) To what extent did teachers believe that they had transitioned from a relative novice in creating NGSS-aligned curricula to an accomplished novice or expert? To what extent were teacher’s curricular and pedagogical shifts in understanding PBL and NGSS reflected in the lesson plans that they developed?
(3) To what extent was the PBL model of NGSS PD useful for teachers? What aspects or features of the PD model were most helpful toward the larger goal of NGSS alignment, and how could it be improved?

We next describe some of the pedagogical shifts mandated by the NGSS, and recommendations for PD as suggested by the relatively young literature on the topic.

The *Framework for K-12 Science Education* (National Research Council [NRC], 2012) provided the foundation for NGSS. Supported by studies in behavioral sciences and science education leading to science education standards and benchmarks developed by the National Research Council (National Research Council [NRC], 1993, 1996), the *Framework* builds on the notion of science learning as a discovery-based and developmental progression, focusing on a limited number of core ideas in science and engineering vital across disciplines. It emphasizes the integration of scientific knowledge (e.g., explanations) and practices of scientific inquiry and engineering design. Integrating research evidence toward a new vision for science education (Metz, 2000, 2004; Lehrer and Schauble, 2002; Schneider et al., 2002), the National Research Council [NRC] (2007) concluded that, with appropriate instruction, students can successfully engage in making hypotheses, gathering evidence, and understanding the phenomenon that they investigate in order to answer complex problems. In addition, they suggested that the teaching of scientific skills is more effectively approached in the context of larger investigations based on questions students help to generate (Collins et al., 1989). Thus, the NGSS has been described as a shift from a focus on learning facts to discovering scientific principles, and from explaining isolated processes to answering larger questions for which those processes are part of the answer (Reiser, 2013).

There is a good deal of evidence that most science classrooms do not engage students in investigating and explaining (e.g., Schmidt et al., 2001; Banilower et al., 2013). Thus, conceptual shifts in the vision for science education mandated by NGSS have placed new demands on teacher learning and PD (Reiser, 2013). This includes shifts from teaching facts to supporting science and engineering practices, and supporting students to explain phenomena (Lehrer and Schauble, 2006). In sum, NGSS was intended to facilitate a pedagogical shift from sequentially pursued topics to lessons driven by questions arising from phenomena, and from testing hypotheses in isolation to investigations guided by explanatory models. The teacher’s role, therefore, is to support knowledge-building practices conducive for forming conclusions and arguments rather than presenting of ideas through lecture and textbooks (Reiser, 2013).

The shift in teaching strategies also involves understanding how different types of science and engineering practices can work together to support a coherent system of investigation and sensemaking. Krajcik (Activate Learning, 2015) emphasized the importance of *three-dimensional learning*, in which *science and engineering practices* (SEPs), *disciplinary core ideas* (DCIs), and *crosscutting concepts* (CCCs), conceived as the three essential elements of NGSS, work together to help learners to make sense of phenomena and design solutions to problems. This approach provides a key emphasis for the aligning of curricular and instructional materials to NGSS. It is also critical for teachers to continually assess three-dimensional learning that occurs in the classroom.

Many teachers who conceptually grasp the curricular and pedagogical shifts involved in aligning instruction to the NGSS still wonder *how* they can make these shifts. Research suggests that teachers struggle the most in the implementation phase when attempting to apply and enact new learner-centered and interdisciplinary pedagogical strategies in STEM (Han et al., 2015). In this project, we suggested to our participating teachers that if NGSS is the “what,” then PBL can be the “how.” That is, PBL can be one solution to the challenge of NGSS implementation. The expectation to emphasize the application of knowledge rather than the recall of facts plays to the strength of PBL approaches. Many of the NGSS standards, particularly the science and engineering practices such as asking questions and defining problems, developing and using models, planning and carrying out investigations, and constructing explanations, align very strongly with PBL (Blumenfeld and Krajcik, 2006; Krajcik et al., 2008). Inquiry, communication, and critical thinking are key competencies both in NGSS and PBL. PBL seeks to provide a meaningful context for the practice of knowledge building and situate scientific ideas through the rich application of problems. These are also goals of NGSS (Krajcik et al., 2008).

Project-based learning is frequently conceived as a model that facilitates learning through sustained work on projects (Thomas, 2000). According to a classic definition, projects are “complex tasks, based on challenging questions or problems, that involve students in problem-solving, decision making, or investigative activities; give students the opportunity to work relatively autonomously over extended periods of time; and culminate in realistic products or presentations” (Thomas, 2000, p. 1). In PBL, students work in collaboration with their peers to define and solve problems, having an opportunity to construct their own knowledge with guidance but not direction by the teacher (Ozel, 2013). However, there is no universally accepted model or theory of PBL.

In this project, we utilized the definition and conceptualization of PBL advocated by the Buck Institute for Education (BIE^1^), widely recognized as providing a “gold standard” vision in PBL. BIE provides the following definition of PBL: “a teaching method in which students gain knowledge and skills by working for an extended period of time to investigate and respond to an authentic, engaging and complex question, problem, or challenge.” BIE emphasizes the importance of a “big idea” or “driving question” around which activities revolve. Krajcik and Czerniak (2007) assert that four critical criteria of good driving questions are that they are: worthwhile, feasible, grounded in real-world problems, and meaningful.

One example of a high quality project according to these criteria is the “Design It Clean” project designed by the New York Hall of Science (2017). In this project, students work in teams to develop water filters designed to be dependable and affordable for the local community. The project addresses the following “big idea” or problem: “Think of the water you used today to shower, cook, or brush your teeth. Although most people in developed nations like the U.S. simply turn on the faucet, approximately three quarters of a billion people worldwide lack access to clean water and millions die each year from causes directly related to this problem.” By asking students to provide clean water for specific communities, they are challenged to ensure that their design meets the needs of the culture, environment, and government of the community. The project is also aligned to NGSS. It applies several science and engineering practices (e.g., constructing explanations and designing solutions), CCCs (e.g., cause and effect), and DCIs (e.g., from molecules to organisms: structures and processes), which can vary depending on the course (e.g., life sciences or environmental sciences).

In addition to BIE’s emphasis on the driving question or big idea in their conceptualization of PBL, they also provide essential project design elements for “Gold Standard PBL” (Buck Institute for Education [BIE], 2017b) based on a review of the PBL literature (e.g., Thomas, 2000; Holm, 2011). These are provided in BIE’s Project Design Rubric (Buck Institute for Education [BIE], 2017a). In this study, we combined the applicable elements from this rubric with the Educators Evaluating the Quality of Instructional Products (EQuIP) Rubric for Science and Mathematics (“EQuIP Rubric for Lessons & Units: Science - Version 3.0,” 2016), in order to analyze teachers’ lesson plans for key characteristics of PBL and NGSS. These key elements of lessons characteristic of PBL and NGSS-aligned curriculum can be found in Appendix A in the Supplemental Materials.

The principles central to NGSS and PBL, especially with respect to conceptualizing learners and the pedagogical shifts that teachers would make to engage them, are highly consistent with those that have been propagated in educational psychology research, broadly conceived as the constellation of research on learning, teaching, and related cognitive, emotional, and behavioral factors. Many of these principles, supported by research and theory in educational psychology over the last century, are summarized in the American Psychological Association’s *Learner-Centered Psychological Principles* (McCombs, 1993, 1997; American Psychological Association [APA], 1997).

Research in educational psychology that support NGSS and PBL have focused on topics such as cognitive constructivism, motivation, and situated cognition (Thomas, 2000). For example, NGSS and PBL activities must involve the construction of knowledge, whereby the learner forms new understandings and/or skills built on previous ones. Characterizing the learning process as one in which the learner actively constructs new knowledge in this manner is supported by research on cognitive constructivism (Piaget, 1968; Schunk, 2008). Research on motivation demonstrates that students with goal orientations focused on obtaining mastery and competency typically enjoy better learning outcomes than those more narrowly focused on their relative performance or social comparisons (Ames, 1992). With their focus on autonomy, collaborative learning, and learning goals, NGSS and PBL maximize student orientations toward curiosity, self-motivation, learning, and mastery. Research on the influence of contextual factors on learning is another area that undergirds both NGSS and PBL approaches. Research on “situated cognition” has identified the importance of real world contexts in which knowledge is used as a key facilitator of learning. Indeed, one inference from this research is that knowledge itself is situated, being part and parcel of the activity, environment, culture, and situation in which it is constructed and used (Brown et al., 1989).

Overall, research has found positive outcomes associated with PBL in a variety of areas. For example, comparative studies have found that students in project-based classrooms made greater gains in content knowledge than in traditional classrooms (e.g., Rivet and Krajcik, 2004; Baumgartner and Zabin, 2008; Geier et al., 2008; Duncan and Tseng, 2011; Kaldi et al., 2011). Students have also reported that PBL fosters engagement and enjoyment of the hands-on approach (Blumenfeld et al., 1991; Meyer et al., 1997; Hmelo-Silver et al., 2007; Baumgartner and Zabin, 2008; Chu et al., 2011; Kaldi et al., 2011; Hugerat, 2016). Students have been shown to effectively learn collaborative learning skills such as perspective taking and conflict resolution through PBL (Chanlin, 2008). Perhaps most comprehensively, one recent random controlled trial randomly assigned 94 6th grade science classes in 43 schools to either a project-based science curricula condition, or one using the district-adopted textbook approach. Students who participated in the project-based curriculum outperformed students in the comparison curriculum on outcome measures and helped students to achieve next generation science learning outcomes (Harris et al., 2015).

In this study, a STEM education center in a large university on the East Coast, U.S. collaborated with a local school district partner to conduct PD for project-based instructional and curricular alignment with the NGSSs. The scope and extent of the project was defined by a grant opportunity internal to the university. The partners co-facilitated a 4-day summer institute and eight monthly PLC meetings designed to help 17 middle and high school teachers adapt, write, and implement NGSS-aligned curriculum and instruction in the context of project-based instruction.

We had several goals for the PD focused on instructional and curricular alignment to NGSS based on a PBL approach. One was for teachers to begin to develop a driving question or big idea, and then drill down into the NGSS standards met, followed by developing more detailed lesson plans. Our approach to the PD was guided by several other aims based on the relevant research to date. One goal was to support teachers in the fundamental shift from director of instruction to facilitator of investigations. Although this shift is easy to comprehend, research has found that it is not always so easy to implement (Han et al., 2015). To sufficiently model this, the PD itself needed to be “learner-centered” to actively engage teachers in PD activities and their own investigative design of new curricula and instructional plans. Due to the interdisciplinary nature of NGSS (i.e., science and engineering practices, CCCs), teachers would also need time to collaborate with each other in order to gain exposure to differences in perspectives and philosophies, and to share expertise, goals, lessons, and timetables. We intentionally provided time and space for this in the PD workshops as well as in the PLCs. Despite the fact that teacher PD frequently utilizes a top-down approach (i.e., university faculty and other experts delivering PD to teachers), expert-centered PD has often been found to be less effective in changing teachers actual instructional approaches than a learner-centered approach (McLeskey, 2011). We also wanted teachers to gain sufficient practical experience with implementation in their own classes. In this project, teachers were encouraged to apply their plans in their classrooms, and obtain feedback from their science supervisor and other PD facilitators during PLCs and in supervision throughout the year.

The project design called for the collection of data to evaluate the PD model, including interviews and lesson plans of six participating teachers. We provide a case of each of these teachers, as well as analyses and conclusions with respect to the research questions in Section “Discussion.”

## Materials and Methods

This study aimed at evaluating teacher participants’ understanding of curricula and instruction aligned to NGSS, and the role of the PD program in aiding in this understanding. A qualitative case study approach provided the opportunity to delve deeply into each case (Creswell, 2007; Yin, 2014), chronicling the journeys and challenges of six profiled teachers as they made critical shifts in their curricular and instructional approach.

### Participants

Seventeen teachers in grades 6–12 were recruited by the school district science coordinator to attend the 4-day summer academy in August, 2015 based on leadership potential (i.e., ability to conduct PD with their teacher colleagues). A small, purposive and ethnically diverse (Caucasian and African-American) sample (*N* = 6) among these PD participants volunteered to provide brief interviews. Of these six, four teachers also provided the curricular unit developed during the PD, utilized as supplemental data for triangulation. Although the remaining two teachers interviewed also wrote new curricula, they did not wish to contribute them. All six of these cases participated in the 4-day institute, and 5 of them participated in PLCs. All of the respondents were female with between 5 and 10 years of prior teaching experience. Three participants taught middle school science and the other three taught high school Biology and Chemistry.

### Procedure

The project PD consisted of a 2015 summer academy and follow up PLCs held approximately once per month during the 2015–2016 school year as described below.

#### Project-Based NGSS Professional Development Summer Academy

The primary goal of the 4-day summer academy was for teachers to learn the basic elements of NGSS and PBL, and to develop an NGSS-aligned unit based in PBL. Primary components of the summer institute were two hands-on engineering design challenges (with emphases on the new engineering practices), a modeling activity, an overview of the critical shifts of NGSS and PBL, a gap analysis to determine holes in existing curricula, and the collaborative development of an original NGSS-aligned instructional unit including a concept map and evaluation rubrics.

On the first day of the 4-day summer institute, teachers were quickly engaged in an engineering design challenge known as “Exploring Buoyancy.” In this challenge, participants use their understanding of buoyancy, density, and pressure to design and build ocean exploring devices (The Tech Museum of Innovation, 2016). Working in small teams, teachers were provided with an array of materials (e.g., balloons, tubing, clay, film containers, paper clips, rubber bands, tape, straws, etc.), and challenged to build a vessel that can float and sink. The vessel had to be controlled from outside of the tank and successfully retrieve a sunken paperclip. Teams were given approximately 40 min in which to build it. Teachers then demonstrated their solution, explained their building strategy, and discussed what they might have done differently. In this activity, participants applied key concepts of buoyancy (including the difference between positive, negative, and neutral buoyancy), density, and the balancing of forces. It meets NGSS standards for grades 3–8, and facilitates familiarity with a variety of vocabulary terms integral to these standards (e.g., Archimedes Principle, Boyle’s Law, equilibrium). The activity also elevates engineering practices, another major goal of NGSS. There were three goals for this first-hand experience with a project-based activity. First, participants were asked to solve a problem with limited directions on how to solve it. Second, participants experienced the need for cooperative teamwork and prerequisite knowledge (e.g., the concept of density). Third, they experienced original and creative thinking as well as frustration with the iterative, trial-and-error design process. These goals were explained in a debriefing that followed the activity.

This activity was followed by an overview of NGSS (e.g., science and engineering practices- SEP, CCCs, DCIs, and three-dimensional learning) and PBL, as discussed in Section “Introduction.”

In the afternoon of Day 1, teachers began a “gap analysis.” They were provided a copy of NGSS standards and performance expectations (PEs). Working independently with assistance from the district science supervisor, teachers identified the standards and PE’s for which their subject and grade band were responsible. Gap analysis refers to identifying the gaps between the current curricula and curricula that would satisfy the standards. Conducting a gap analysis involved a good deal of complex re-thinking of how curricula and instruction are designed. As teachers completed this task, they began to think of driving questions or big ideas that would be ideal for teaching the standards for which they were responsible. This then led to “bundling” a collection of standards that could be explored together in the investigation of a given driving question or subordinate guiding questions. Teachers then mapped these bundles and possible projects onto specific courses and units.

On Day 2 of the institute, teachers participated in another engineering design challenge called the “Toxic Popcorn Design Challenge” (“Try Engineering - Toxic Popcorn Design Challenge,” 2017). The challenge of this activity was to develop a process and product to transfer toxic popcorn to a safe location in order to save a city. This design challenge was similar in its goals to the “Exploring Buoyancy” challenge.

Following this, teachers were engaged in a modeling activity intended to be a strong example of an NGSS-aligned unit and three-dimensional learning. The investigation revolved around the cause of a worrisome quantity of fish discovered dead in a local pond. Teachers were asked to conjecture their hypotheses. They were also provided with a variety of data sources that support some causal explanations while weakening others. Participants were asked to construct causal models using an online modeling tool called “Ecomodeler”^2^ to provide a visual representation of their models with easy incorporation of new components or patterns observed with additional data. Generally speaking, participants built increasingly sophisticated and complex models as the activity continued, allowing for the testing of more sophisticated explanations. This activity demonstrated the understanding and use of a variety of SEPs (e.g., planning and carrying out investigations, developing and using models, conducting observations, engaging in data analysis and interpretation, and developing explanations); CCCs (e.g., cause and effect, patterns, systems and system models); and DCIs from the physical sciences (e.g., matter and its interactions, energy) and life sciences (e.g., ecosystems).

Following the modeling activity in the afternoon of Day 2, teachers continued to work in pairs (based on subject and grade) and continued their mapping and bundling task. As soon as a basic skeleton of units and lessons was developed, the pair transitioned into a more detailed design of lessons in the unit. Teachers were encouraged to continue making links among the driving question, standards, PEs, and instructional activities.

The balance of the third and fourth days of the institute was spent continuing this work. Very little direction or unsolicited scaffolding was provided. Rather, facilitators observed, supervised, coached, and were available to assist with this work as needed. A great deal of teacher PD has been found to be relatively ineffective when it is expert-centered and dominated by direct instruction (Han et al., 2015); therefore, we intended to take an explicitly project-based approach in modeling the PD instruction. Teacher participants were free to design units and performance-based assessments in ways that were the most useful for their particular grades, subjects, classes, and students.

By the end of the summer institute, all participants had successfully conducted a gap analysis, bundled a collection of standards with driving questions, mapped them to specific courses and units, and developed the basic structure of specific units and lessons. The extent to which teachers were able to design assessments with the time available varied. It was understood that teachers would continue to develop, detail, and pilot their lessons throughout the school year.

#### Curriculum Writing

All project participants were instructed early in the 2015/2016 academic year to develop new NGSS-aligned curricula, and to share their curricula documents with their district science coordinator and each other via Google documents. Teachers were also encouraged by their district coordinator to keep a journal or log notes on the progress of curricula implementation, and to save examples of student work for sharing in PLCs.

#### Professional Learning Communities (PLC)

Professional Learning Communities meetings were co-facilitated by the university center staff and the district science coordinator. All study participants attended the PLCs except for one. Typically, 2–4 teachers attended a PLC meeting. Each grade was assigned a teacher-leader who created NGSS-aligned lesson plans and shared the most updated one with other teachers in the same grade band during the PLC. During the meetings, the other teacher participants also shared their experiences with implementing their NGSS-aligned curriculum in their classrooms. The group discussed practical difficulties and successes with their curriculum design, and challenged each other’s ideas and implementation strategies. They also compared and discussed NGSS-aligned assessments and rubrics. Facilitators provided insights and feedback on pedagogical strategies, helped to evaluate student projects, served as a sounding board for new iterations of the curricular units and engineering challenges, and addressed specific challenges associated with each lesson.

Due to the state mandate to begin implementing NGSS starting the fall of 2016, teachers were generally receptive to the PD and the PD facilitators.

### Data Collection

Data collection included teacher interviews of six teacher participants and the collection of lesson plans for the four of these participants for which they could be obtained.

#### Teacher Interviews

Interviews were administered following the summer academy and during the academic year after teachers participated in the PLCs. Conducted by one of the university research associates, the interviews posed questions to volunteering teacher participants about their current understanding of NGSS, their self-perceptions about their level of accomplishment as NGSS-aligned curricula designers, what had been gained through the project PD, and suggestions for PD improvement. Interviews lasted approximately 20 min. The interviews were audio recorded and transcribed verbatim.

Our interview protocol, structured around the three research questions, is presented in **Table 1**.

**Table 1:** Interview protocol.

Interview item
(1) What prior experience did you have related to NGSS before the project began? (RQ1)
(2) What do you think are the most important or critical aspects of NGSS and why? (RQ1)
(3) What topic did you choose for the lesson you created and aligned to NGSS and why? (RQ1)
(4) What were the major ideas or aspects of NGSS that you incorporated into your lesson? (RQ1)
(5) How has your understanding of NGSS and the content been influenced by working on the curricular alignment? (RQ1)
(6) What prior knowledge did you draw on in writing your curriculum? (RQ1)
(7) What assessments did you use to evaluate student understanding? (RQ1)
(8) What were your greatest challenges in writing new curricula? (RQ1)
(9) Do you think of yourself as a novice, accomplished novice, or expert? (RQ2)
(10) Was the project PD (i.e., summer academy and PLCs) helpful? (RQ3)
(11) What was the most beneficial part of the summer academy? (RQ3)
(12) What was the least beneficial part? (RQ3)
(13) Was there anything that the PD could have included that would have been helpful, but didn’t? (RQ3)
(14) What sort of preparation would have you undertaken if you did not have the opportunity to participate in the project PD? (RQ3)
(15) Based on your experience with the PD, how confident do you feel in writing NGSS-aligned curricula, and in helping other teachers to do so? (RQ3)

Questions 1–8 were designed to probe the respondent’s current understanding and appreciation for NGSS, the curricular and pedagogical shifts necessary to comply with NGSS, and the current status in the teacher’s journey toward conversion to NGSS from a variety of angles (to answer research question 1). Question 9 asked teachers their self-perception of their transition from novice to expert, which we triangulated with an analysis of each teacher’s lesson plan (research question 2). Questions 10–15 asked participants to reflect on the perceived benefit of the project PD and possibilities for improvement (research question 3).

#### Teacher Lesson Plans

Lesson plans were analyzed with an applicable NGSS and PBL lesson plan rubric that the research team created by fusing together (a) relevant elements of NGSS alignment from the Educators Evaluating the Quality of Instructional Products Rubric for Science and Mathematics (“EQuIP Rubric for Lessons & Units: Science - Version 3.0,” 2016), and (b) essential elements of PBL from the Buck Institute for Education’s Project Design Rubric for (Buck Institute for Education [BIE], 2017a). The elements in this combined rubric were: (1) Key Knowledge, Understanding, & Success (i.e., clear learning goals aligned to standards, BIE); (2) Challenging Problem or Question (guiding question or big idea, BIE); (3) Sensemaking (i.e., making sense out of phenomena and/or designing solutions to a problem to drive student learning, EQuIP); (4) Alignment to NGSS Core Components (i.e., opportunities to develop and use SEPs, DCIs, and CCCs, EQuIP); (5) Integration of the Three Dimensions of NGSS (i.e., sensemaking and problem solving with SEPs, DCIs, and CCCs, EQuIP); (6) Development of Performance Assessments (eliciting observable evidence of three-dimensional learning, EQuIP); (7) Sustained Inquiry (BIE); (8) Authenticity (i.e., situating instruction in real-world contexts, BIE); and (9) Monitoring Student Progress, Feedback, and Revision (i.e., authentic assessments, EQuIP; feedback and opportunities for revision, BIE). The full rubric is presented in Appendix A in the Supplemental Materials. Another framework for analyzing lesson plans came from the “learning-goal-driven approach,” which also fuses NGSS alignment with PBL, developed by Krajcik et al. (2008). These tools were utilized for internal analytic purposes only. We lacked sufficient information to reliably rate the lesson plans on every element of the rubric; for some elements, this would have required samples of instruction, not just the lesson plan. Rather, inferences from the analysis are reported descriptively in Section “Discussion.”

### Data Analyses

The present study utilized a qualitative, ground-up approach for analysis. Thus, characterizations of teacher’s conceptual and curricular shifts were rooted in participants’ *own descriptions.* We then sought to discover patterns as we looked across participants to answer the research questions. In addition, we leveraged additional information from the lesson plans where available to provide a different perspective on the level of understanding obtained by the participants in writing NGSS-aligned curricula.

Data was analyzed by three experienced members of the research team from the university STEM education and research center. The research team wrote a case study for each participant based on the interview and lesson plan if available (Yin, 2014). These are presented in Section “Findings.” The team then met to discuss patterns and form consensus regarding the answer to research questions across participants (Denzin and Lincoln, 2005; Creswell, 2007). We developed a systematic and organized process for the emergence of themes (Glaser and Strauss, 1967), developing several tools to help analyze the data. For research question 2, related to teacher accomplishment in designing NGSS-aligned curricula, this included the creation of the NGSS and PBL lesson plan rubric, and its application to the teachers’ lesson plans. These tools were jointly applied, and conclusions were reached by consensus. Themes and conclusions are presented in Section “Findings and Discussion.”

## Findings

Case studies of each respondent are provided below. All names are pseudonyms, and specific subject and lesson topic information are masked to protect subject anonymity.

### Case 1: Cecily

Although we had assumed that all of the teacher participants in the NGSS PD were complete novices when the PD began, Cecily was an example that this may have been an oversimplification. She traced her introduction to NGSS back to 2012, 3 years before the project year. Even though this earlier training was not formally focused on NGSS, she felt that the overlap was significant in terms of the influence in her thinking processes. Subsequently (but still before the present PD project), she started using NGSS standards even though they were optional. She repeatedly expressed the ways in which her background stemming back to graduate school provided an important frame of reference and level of comfort for understanding NGSS. Nevertheless, she also clarified that she progressed significantly since the beginning of the project, and that using the standards in the current year was key to her current state of development.

In discussing assessments, she demonstrated her appreciation for PBL as a key strategy for approaching NGSS: “(For) all of the units, their major assessment is basically to have a project. And the way that I’ve set up the project on the rubrics is basically looking at the standards and aligning it to a task that they’re able to complete as a component of the project or as a project in itself.” Cecily appeared to be expressing advanced understanding of NGSS and PBL integration as outlined by Krajcik et al.’s (2008) learning-goals-driven model, in which assessments and rubrics are thoughtfully aligned to learning goals and performances.

Cecily identified several challenges in her journey toward implementing the full set of NGSS standards. She felt that a primarily challenge was having the time for lesson planning and curricula writing. She also found that far from “being done” after writing a lesson, NGSS curricula was more of an iterative process due to her perceived need to continually go back and make adjustments in her lessons. With respect to classroom teaching, she found that her content knowledge was being pushed, sometimes teaching content that she learned only the day before. This seemed to be due to the fact that content was no longer taught in a narrow and circumscribed way; in a broad investigation, a larger set of scientific principles and knowledge could be applied. As she put it, “I don’t know everything about everything.”

Overall, Cecily considered herself to be an accomplished novice. She did not consider herself to be an expert in writing NGSS-aligned curriculum since it was still a new activity. However, she noted that her work on aligning assessments was being used as a model for other grades, and that she was very proud of that. At the time of her interview, she was feeling “confident” in her ability to conduct teacher PD with her colleagues, or at least to “assist” them.

Cecily designed several NGSS-aligned units over the course of the year. The research team concurred that her lesson plans demonstrated a high level of understanding of NGSS and NGSS-aligned curricula, as well as PBL, on most of the indicators in our NGSS-PBL lesson plan rubric (represented in the third column of Appendix A of the Supplemental Materials). Her units revolved around driving questions meeting Krajcik and Czerniak’s (2007) criteria for good guiding questions (i.e., worthwhile, feasible, grounded in real-world problems, and meaningful). Her lessons were aligned to numerous, appropriate DCIs, CCCs, and science and engineering practices (although notably more science than engineering practices). In general, her units were not only well-aligned to NGSS with respect to framing full investigations that facilitate three-dimensional learning, but they also demonstrated an excellent command of PBL in terms of situating her units and lessons in strong driving questions and real-world contexts.

Cecily reported that the PD program – both the summer institute and the PLCs – were very helpful in her journey toward NGSS alignment. The part of the summer academy that she found the most helpful was seeing engineering-type lessons and experiencing them as a participant. She recalls:

I remember the activity with the buoyancy where we had to make it sink and float back up and pick up the paperclip. And I looked at it and I was like, ‘Oh my god there’s no way I can do this, this is so challenging.’ And then we worked together, we figured it out, and we made it work and I was like so proud and I was like, ‘Oh this is totally doable, our kids can do this too.’

The other aspects that she enjoyed were time to work on the curriculum and interacting with teachers of other grades and subjects. She particularly appreciated talking to teachers of other grade bands working on similar subjects and units. This helped her to understand the “vertical articulation” with respect to progressive expectations in standards and performances as students developed and progressed through the grades. Cecily found it helpful to collaborate with colleagues during the PLCs as well as during common planning time. Colleagues were able to share activities, differences in implementation, and progress, which she found varied from classroom to classroom and unit to unit.

Cecily found that there were no parts of the PD program that were not helpful. She identified CCCs and how to incorporate them into lessons as one challenging topic on which she could use more guidance, however.

### Case 2: Caroline

Caroline believed that her prior teaching experience was the most influential factor in her ability to create new NGSS curriculum units. According to Caroline, the most critical aspect of NGSS is the underlying themes as represented by the CCCs. Because science concepts are interrelated rather than isolated, the CCCs are important to tie them together. She said “they build upon one another so it connects the dots for our students.” Also, in her opinion, NGSS gives teachers more freedom and fewer restrictions.

In speaking about her new curriculum units that were intended to align to NGSS, Caroline said that she tried to include more inquiry-based learning and to make the activities more investigative. She tried to incorporate projects in order to minimize the amount of isolated activities and facts. Caroline indicated that including projects made the learning more meaningful. In general, she attempted to move the units from being teacher-centered to student-centered. In terms of assessments, Caroline attempted to utilize PBL and have students write up lab reports. Students were given formative feedback on their lab reports. She also identified the use of creative projects for having students make claims, provide evidence, and use reasoning to answer some driving questions.

Caroline explained that the biggest challenge she faced was trying to incorporate engineering practices. Prior to starting her unit, she spent class time trying several engineering activities and demonstrating the engineering design process. She found that it required a good deal of time and delayed implementation of other units.

Overall, Caroline considered herself to be an accomplished novice. She did not consider herself an expert yet because she believed that there was still much to learn. When asked, however, Caroline stated that she was confident enough with NGSS to train other teachers.

Her lesson plans, however, suggested that she was assigning new standards to her previous curricular units rather than rethinking the units from an entirely new and NGSS-aligned perspective. Her lesson plan did not align strongly to NGSS with respect to framing a full investigation that facilitates three-dimensional learning. Some of her driving questions were meaningful and worthwhile, but not feasible or based in real-world contexts. Others were feasible, but did not meet the other criteria for good driving questions. They remained academic and isolated from real-world problems. Main activities were reading, think-pair-shares, and writing; the lesson plans included very little interactivity or collaboration with some exceptions. Overall, her unit would need to be considerably reworked in order to exemplify NGSS alignment. The research team agreed that her lesson plans demonstrated a minimal to partial understanding of NGSS and NGSS-aligned curricula based on most of the indicators in our NGSS-PBL lesson plan rubric (described in the first two columns of Appendix A of the Supplemental Materials).

Caroline believed that she benefited from the project PD. She felt that learning to incorporate the PBL approach into NGSS-aligned lessons was the most valuable part of the summer academy. She also appreciated time devoted to unpacking the standards. She stated that without the summer academy, she would have been overwhelmed. Caroline stated that there were no parts of the PD program that were not helpful, but that she would liked to have seen more examples of NGSS-aligned lessons.

### Case 3: Janet

Janet stated that the summer academy provided her with background knowledge of the core elements of NGSS. This knowledge was strengthened by collaborating closely with another high school teacher during the summer academy, during the PLCs, and beyond.

Drawing from her teaching experience, Janet emphasized the importance of student engagement on learning. She believed that a high level of student engagement on select topics was more valuable than covering a wide breadth of topics and superficially touching upon multiple science standards. She believed that NGSS would help students to step away from rote memorization and instead concentrate on the processes of “doing science” through the science and engineering practices. She stated that her intent in designing curricula was to explore the CCC of modeling through model creation, helping students to rely on models to make sense of phenomena and to evaluate them. In terms of challenges, she admitted that she was not confident of how to align engineering activities with content standards in her subject.

Janet reported that she felt to be an accomplished novice and an expert as a result of her experiences at the summer academy and PLCs. In reviewing her unit design and lesson plans, it was clear that she expected students to plan and conduct investigations, and rely on scientific evidence to support claims. Importantly, her lessons intended for students to consider CCCs such as engaging in systems thinking. It could also be observed that she attempted to incorporate some elements of the engineering practices. Her curriculum also illustrated an instructional process by which she engaged students to create and work with models. Students were expected to revise their models collaboratively based on class discussion and evidence from labs, which were carefully designed to influence students’ models.

Overall, the consensus of the research team was that Janet demonstrated a high level of understanding and application with respect to most of the indicators on our NGSS-PBL lesson plan rubric (i.e., third column in Appendix A of the Supplemental Materials). For example, each of her lessons extended and enriched sustained inquiry in a series of discovery-based activities investigating her driving questions. She also utilized authentic assessments.

Janet reported that she found the project PD helpful; she was grateful for the experience and how much she learned from it. For her, the most helpful aspects of the summer academy were the time to write new curricula, the grouping of standards, and the mapping of standards to curricula. She reported that there were no parts of the PD that were not beneficial, although it would have been helpful to see more models of NGSS-aligned lessons and assessments. Had she not participated in the PD, she believes she would have tried to “shoehorn” the NGSS standards into her current curricula and instruction and previous way of teaching; through the project PD, however, she understood that NGSS alignment entailed a shift to a completely new way of curricula writing and teaching. She felt confident in her ability to help other teachers work with the new standards.

### Case 4: Heather

While the summer academy was Heather’s first exposure to NGSS, she reported having developed student-led, PBL activities as part of her pedagogy in the past. As a result, she was excited to explore the extent to which she could redesign her prior lessons by aligning them to NGSS.

Factors that influenced Heather’s unit design were a combination of her academic background and prior classroom teaching experience. She relied on her strong content knowledge to identify specific DCIs that would be central to her unit. Having taught several NGSS core concepts in the past, Heather was sensitive to students’ interests and common misconceptions. She anticipated assigning her students to work on multiple projects, with experiments, labs and modeling as key elements. She envisioned assessments based on students’ abilities to create and analyze models. The biggest challenge Heather faced was lacking enough time to implement the unit before the end of the school year.

Heather felt that she had a strong understanding of NGSS disciplinary content areas. Her primary take away from the summer academy was the notion that NGSS encouraged designing “encompassing projects or topics” for real-world problem solving. Overall, she felt that she was not an “expert,” but that the PD prepared her to have a good understanding of various facets of the NGSS as an accomplished novice.

Her lesson plan focused on students’ engaging in scientific practices of argumentation, and analyzing and interpreting data by working independently and in collaboration. Other than this, however, they were heavily content-based. The essential questions were mainly academic, cut off from real world application, and did not meet any of Krajcik and Czerniak’s (2007) criteria for good guiding questions. The research team concurred that her lesson plans demonstrated low to moderate understanding of NGSS and NGSS-aligned curricula based on most of the indicators in our NGSS-PBL lesson plan rubric (i.e., first two columns of Appendix A of the Supplemental Materials). Some of the activities were designed to be active, such as lab work and annotating articles; students were also to spend a good deal of time making posters. This was followed by sharing findings in groups. She also incorporated demonstrations designed to provide first-hand encounters with phenomena that would spark interest and curiosity. This was mixed with lectures, overhead projector slides, videos, and other forms of direct instruction. Most of the activities remained somewhat procedural and detached from any larger investigation or sustained inquiry that would tie the activities together. The posters were to present results of labs on circumscribed and narrow questions. The lesson plans would have been improved by spending more time on inquiry in the context of a fuller investigation driven by an authentic, real-world question.

Heather also found the project PD beneficial. Aside from being exposed to hands-on activities, Heather was grateful for the time and opportunity to “sit and think and brainstorm ideas and to write out lessons.” She found it particularly useful to have the time to ask questions to her colleagues. She also found the whole group activities at the beginning of the academy to be helpful with respect to making sense of engineering practices. She incorporated one of the activities in her own classroom so that students would understand that “science is all about solving the world’s problems.”

While she was unable to identify areas for improvement of the summer academy, she did anticipate several areas for personal improvement. She believed that she would achieve improvement by “trial and error, and keeping up with the standards.” In addition, she hoped to refine her lesson plan by seeking feedback from the science coordinator and members of the project team.

### Case 5: Jennifer

Jennifer’s case study was the least instructive. In addition to lacking her lesson plans, she provided only very brief and cursory responses to the interview questions. She felt that the NGSS standards are definitely important in terms of the science concepts that they embody, and because they help to organize them for teachers. She reported that there is almost no connection at all between her existing curricula and her present plans to align with NGSS. However, her responses were unconvincing with respect to making a meaningful curricular or pedagogical shift during the project period. She attempted to use open notebooks and projects for assessments. She felt that among the greatest challenges was “getting it across to the students.” Overall, she considered herself “accomplished but not an expert” at aligning curricula to NGSS.

She did believe to have benefited from the PD, finding that the most beneficial aspects were the hands-on activities and the web resources made available. There was no part of the summer academy that she did not find helpful.

### Case 6: Kate

Although we also lacked lesson plans for Kate, her interview was very instructive. She expressed a belief that the SEPs, DCIs, and CCCs are equally important. She understood that these dimensions working together in three-dimensional learning are designed for learners to develop deep understanding and the ability to apply it. She saw the conversion to NGSS as a “total transformation.” She found almost no connection at all between her existing curricula and her present plans to align with NGSS.

Appreciating the difference between understanding and application, she stated, “It’s not enough to memorize.” As she worked on new curricula with a teacher colleague, she reflected,

It’s like being a brand new teacher all over again and then it’s content that I don’t even have a degree in. So she and I both have felt very much like we’ve been in deep water all year. And we sort of have had to say, ‘You know what this is a great idea, this is a wonderful thing, we love NGSS, but we are going to need years to develop this.’ NGSS isn’t just content, I can’t just show a PowerPoint to my kids and be doing the NGSS.

Nor, she found, could she just test students to see how much they know. Thus, she has had to learn “a whole new way of doing things,”

With respect to a project-based approach, she stated, “I love the idea of the entire course being a series of projects and if I had projects that I could use that were at an appropriate level then I would just jump in with that because I love the idea of having to figure stuff out.” However, she was realistic about the inherent challenges to high quality PBL instruction. For example, she felt that PBL requires a great deal of scaffolding, and that this requires so much time that there was not always the time to model the scientific method thoroughly. Ultimately, she believed there was a trade-off between being able to engage in PBL and covering all of the NGSS standards for which she was responsible.

Kate expressed both a deep appreciation and real concern for numerous other challenges involved in trying to implement NGSS. First, once the PEs were divided among the school subjects in her school, she found that there was a lot of new content that she had to learn to cover them. Secondly, she was reluctant to state that she has been able to fully meet certain standards. For example, in conducting a modeling activity, she observed, “I’m not sure we got to the point where the kids were developing their own models.” Kate detailed the vast amount of scientific knowledge that was rolled into a single PE, declaring, “So it felt like there were so many parts; I really feel like each PE could be its own unit.” This challenge, in turn was related to the amount of instructional time required. She felt overwhelmed by the large amount of time that would be required to teach multiple standards due to the richness of each.

Kate found that because NGSS is new to students, they have little prior background (e.g., CCCs) and few of the competencies (e.g., SEPs) that are the focus of NGSS. This also contributed to the requirement of greater instructional time. Lessons she had planned to take 1 day required at least 2 days, and that a 10-day unit “turned into a 23-day unit.” Thus, she found that a real challenge was making NGSS “fit” into the constraints of public schooling. Her perception that it would take years to develop NGSS lessons well was supported by the observation, “It would be hard enough to do if we were using a packaged curriculum and just trying to be the facilitators of that with the kids, but the fact that we are coming up with every activity, every assessment, every project, every lab ourselves, and then trying to adjust it to our personal styles, that just adds a whole other layer.”

An additional challenge was lack of qualifications in some of the advanced reasoning skills that teachers are now expected to teach students. She felt that she and her teacher colleague “weren’t necessarily comfortable with claim/evidence/reasoning and we certainly didn’t have any experience teaching it.” She admitted that she was not sure if anyone appreciated just how content-driven science instruction has been up until this point. Kate recognized that she would still need a great deal of support, and was concerned with her ability to obtain it. She stated that she got a 4-year degree in her subject area, not a 4-year degree in how to teach it. Implicit in this statement is the perceived need of an entirely new and different pre-service education focused on pedagogy in order to meet the pedagogical demands of NGSS. She admitted to feeling overwhelmed by such a large change.

Kate stated that she did not perceive herself as even a novice yet, but felt to be a complete newcomer. She explained: “I would need an entire course in just what each standard really contains…I could literally spend a number of hours delving into how all the parts come together to make one PE and then how to get the kids to be able to do that….I don’t know if I accomplished what was expected. This whole year was a year of not getting there.” Toward the end of the interview she openly confessed, “I don’t feel qualified to teach NGSS to students.”

With respect to the project PD, Kate stated that it “helped to see the richness” of NGSS, and that NGSS “is not like anything we’ve done before.” She also believed that it was very helpful to coordinate expectations with her science supervisor and teacher colleagues. One of the most helpful aspects of the summer institute for Kate was doing design challenges as a student; however, she felt that it would have been even more helpful if the PD had come full circle and she had an opportunity to then learn how she was supposed to implement the activity as a teacher. She reported that there was nothing about the summer academy that was harmful or detracting. She also shared that she didn’t know what she would have done to prepare for NGSS without the project PD.

## Discussion

This study sought to present a model of NGSS PD emphasizing a PBL approach consistent with the principles of NGSS in order to provide teachers with the support needed to write NGSS-aligned science curricula. We then attempted to evaluate the extent to which teachers understood core principles of NGSS and were able to make the curricular and pedagogical shifts necessary to align with NGSS. We also assessed degree to which the PD model was beneficial to teachers and could be improved. This study thereby contributed to knowledge about teachers’ experience and self-perceptions in transforming their practice to project-based methods aligning with NGSS.

### Teachers’ Shifts in Their Journeys toward NGSS Alignment

When the summer institute began, most teachers had a strong background in their core scientific area, but all were novices in NGSS curriculum development. However, there was more variation than anticipated in teacher’s prior experience that was relevant to the understanding and application of NGSS. Prior exposure and experience related to NGSS appeared to be a highly relevant factor in determining the extent of teachers’ shifts as they interacted with NGSS PD. Thus, the assumption that all teachers enter NGSS PD as complete novices was shown to be an oversimplification.

All of the teachers in this study demonstrated some measure of conceptual understanding of the core components of NGSS. Some of these conceptualizations were quite complex and advanced; some were more rudimentary. All of the teachers in our study also articulated some aspects of NGSS that they believed to be important. The diversity of NGSS characteristics identified as valuable included the emphasis on students “doing science,” CCCs, teacher autonomy, learning by doing, PBL, the organization of important science concepts, and the depth of learning produced by thinking in the three dimensions of NGSS.

Most (i.e., five out of six) of the teachers in our sample articulated significant curricular and pedagogical shifts that they had undergone following the summer academy and as they participated in follow-up PLCs. As a group, teachers understood the value of providing projects, and contextualizing problems as context for students’ “need to know” science concepts (Edelson, 2001). They believed that this shift in pedagogical practice from direct forms of instruction made classes more interesting for students. Instead of a focus on memorizing scientific facts, students were now expected to focus on the process of scientific inquiry and problem solving through projects and engineering design challenges.

The nature of their shifts and extent to which the project PD influenced them varied, however. For some teachers, the PD provided the necessary background to understand the core elements of NGSS. For others, the PD helped to support shifts from teacher-centered to student-centered or inquiry-based pedagogy. One teacher provided an expanded articulation of the “total transformation” that she believed was now necessary. For her, there was a great deal involved in designing the conditions for students to make their own connections and build knowledge through discovery and reasoning. She embraced the emphasis on learning through a series of projects, but also realized that the degree of scaffolding and support necessary to do this well would soon outstrip available time. She felt that the transition to NGSS marked a “completely new way of doing things.”

### Teacher Challenges in Writing and Implementing NGSS-Aligned Curriculum

The challenges and obstacles encountered by teachers as they transitioned to the new standards also varied, but there were several common themes. The most common challenge mentioned was the need for more time – both planning as well as instructional time. For example, some teachers reported that teaching engineering through the types of engineering design challenges introduced in the PD required significant time, delaying the start of other units. For one teacher, delivering instruction tended to take twice as long as planned, competing with covering other standards and PEs.

Another challenge area that emerged was lack of adequate knowledge and skills required, for both teachers and students. For example, one teacher was not completely comfortable with the teaching of advanced analytic skills such as supporting claims with evidence. Meanwhile, other teachers identified students’ dearth of knowledge of core NGSS components as a significant challenge, due to the lack of exposure in previous grades.

### Teachers’ Level of Accomplishment from a Relative Novice with NGSS

Although most teachers had started the PD program as relative novices, five out of six reported feeling like “accomplished novices” by the end of the program. These teachers also felt confident in their ability to teach their colleagues how to align curricula to NGSS. One of the teachers, however, described herself as barely novice or a complete newcomer. She believed that she was “unqualified” to teach NGSS. This teacher was extremely articulate in her understanding and appreciation of NGSS, and believed that her shift was a total transformation. Therefore, this appeared to be a case of, “the more you know, the more you know how much you don’t know.”

We analyzed the lesson plans provided by four teachers, all of whom perceived themselves to have made significant curricular and pedagogical shifts. We found that the level of understanding and application of NGSS demonstrated by teachers’ lesson plans among this group ranged significantly, and was not consistently related to teachers’ self-perceptions. The lesson plans of two of the teachers identified good driving questions and clear learning objectives aligned to standards, and designed a series of investigations and learning activities illuminating different aspects of those questions in which students applied targeted concepts. However, the lesson plans of two teachers met such criteria only inconsistently or in a very limited way. For example, they did not incorporate key aspects of science and engineering practices.

Several study limitations may temper these results. First was the very limited sample size which limits the capacity to generalize from the findings. Notably, only six of our 17 PD participants provided the case studies that led to study findings. Second, there may have been a response bias stemming from the fact that two of the teachers did not contribute a lesson plan. Nevertheless, we can infer from the small sample size a *minimum* amount of variation in teachers’ demonstrated level of competency in curricular alignment following PD designed to support it. We found that this minimum variation was not trivial. In practical terms, some teachers may be quite proficient at designing aligned curricula following as little as 4 days of intensive PD and follow up PLCs, especially those who have relevant prior experiences. Other teachers are likely to need additional support.

### Barriers to NGSS Implementation

This study suggested numerous potential barriers to NGSS implementation. Both the challenges that teachers described, and the variation in proficiently aligning curricula following the PD, can be beneficially viewed from the perspective of implementation science and design-based implementation research (e.g., Klein and Sorra, 1996; Dane and Schneider, 1998; Carroll et al., 2007; Damschroder et al., 2009; Penuel et al., 2011). Implementation has been defined as “the constellation of processes intended to get an intervention into use within an organization” (Damschroder et al., 2009, p. 3). At the larger level, NGSS can be viewed as a type of intervention intended to change behavior within an organization; and on a smaller level, the PD program was a type of intervention to facilitate NGSS adoption.

Based on research in health services and school psychology, factors influencing the implementation of interventions include characteristics of (a) the intervention, (b) the inner and outer setting, (c) the individuals involved, and (d) the process by which the intervention occurs (Damschroder et al., 2009). Characteristics of the *intervention* include its source, and evidence strength and quality. From the perspective of schools, NGSS can be considered an externally developed reform effort, but one in which the quality of the evidence base is generally considered to be high. Two other important aspects of the intervention predicting the success of implementation are its *complexity* and *adaptability*. NGSS calls on teachers to model and students to engage in “three-dimensional thinking,” which demands a variety of aptitudes to make numerous complex decisions, and many other skills to implement them effectively. Most interventions in the health care field are much simpler by comparison – and they are implemented with mixed success. Because teachers have great leeway to design and implement in their own way, NGSS is also infinitely adaptable. It is adaptable, however, partly by virtue of espousing general principles instead of recommending specific actions. The immense complexity and adaptability of NGSS can be overwhelming, as expressed by one of our participants. Thus, the health care field might view chances of successful NGSS implementation as unrealistic based on its complexity and adaptability.

Characteristics of the *setting* have been conceptualized by a distinction between the inner and outer setting. The outer setting includes the economic, political and social context in which an organization resides, and the inner setting includes structural, political, and cultural contexts through which the implementation proceeds (Pettigrew et al., 2001). The most salient aspect of the outer setting for NGSS may be external policies, i.e., the state adoption of NGSS and expectations for implementation. This may be supported by an element of peer pressure (i.e., other states and schools adopting and implementing NGSS). The inner setting includes structural characteristics, networks and communications, and culture. Most relevant to NGSS, however, might be aspects of *implementation climate.* This includes receptivity to involved individuals, which we have observed to be relatively high. It also includes *compatibility*, or the extent to which the intervention fits with existing norms, values, workflows, and systems (Klein and Sorra, 1996; Greenhalgh et al., 2004). For schools, characteristics of compatibility include structural features such as the school schedule and the time and necessary education afforded to teachers who implement the NGSS. According to one participant, these aspects of the inner setting posed some of the greatest challenges to implementation. It is not difficult to conclude that although NGSS is well-intended and generally well-received, it also sets up a clash between the inner and outer setting. Ultimately, its success may reside in another feature of the inner setting, which is its level of *commitment*, where commitment is defined by making modifications necessary to successfully adopt the NGSS and meet the demands of the outer setting. Our study would suggest that such commitment would include a reduction of coverage expectations, and additional provisions for planning and instructional time. Treated more thoroughly, it might include a re-conceptualizing of the school day or the extent to which teachers co-plan shared curricula or projects beyond their individual classes.

Characteristics of the *individual* include knowledge and beliefs about the intervention, necessary competencies and background, self-efficacy, and the individual’s stage in the change process (Damschroder et al., 2009). As we have noted, background and stage in the process appeared to play a large factor in explaining variation among teachers in their ability to align curricula. Even though on paper all teachers were assumed novices, some teachers had greater familiarity with the changes sought, positioning them at a more advanced stage. However, we do not know the extent to which teachers’ ability to align curricula could also be related to other characteristics such as their affective response, investment, and willingness to embrace NGSS. Future studies may probe this topic further.

Characteristics of the *process* include planning, engaging in, executing, and reflecting on the intervention (Damschroder et al., 2009). It was the process of implementation that our PD program was designed to influence. We acted as external change agents by providing a model of NGSS, and afforded opportunities for reflection on the process by hosting PLC meetings. In focusing on curricula design, we targeted the planning part of the process specifically. Executing the intervention with *fidelity*, or as it was intended, is another critical aspect of implementation (Carroll et al., 2007). In our study, we did not focus on or evaluate classroom implementation. However, in evaluating curricula alignment with EQuIP and PBL rubrics, we evaluated the fidelity of teachers’ *plans* for implementation with the essential elements of NGSS and PBL. Some participants in our study believed that they had benefited and grown from the PD, but did not write aligned curricula as a result. This suggests that to evaluate PD effectiveness, fidelity to the tenets of the PD in executing curricula and instruction must be considered.

There is evidence that greater complexity of interventions inhibits fidelity (Dusenbury et al., 2003). Our PD program was intended to simplify the complexity of NGSS by providing problem-based learning as a model and facilitation strategy. As one of our case studies vividly illustrated, the great complexity of NGSS raises the important issue of *adequacy* of teacher education and PD for NGSS to succeed (Carroll et al., 2007). Related to adequacy of PD, the frequency, duration, and sustainability of content coverage (i.e., “dosage”) have also been identified as critical features in effective PD (Joyce and Showers, 2002; Mergendoller et al., 2006). There were suggestions that a 4-day workshop does not meet a reasonable criterion for adequacy for many teachers. Thus, PD of greater duration is one recommendation for future NGSS PD. Another recommendation is the complexity of implementation be moderated by teachers’ level of experience or stage in the process of change. For less experienced teachers, curricular writing may be broken into several discrete parts; or they might be asked to adapt and align curricula with which they are already comfortable before designing new or original curricula.

### Benefits of the PD and Suggestions for Improvement

All of the teachers in the study indicated that the project-based PD model was beneficial to their development. Several teachers shared that their existing content knowledge supported only a fraction of what is required for NGSS-aligned curricular design and implementation. For example, many teachers’ knowledge of CCCs and engineering practices were not adequately developed in order to create NGSS-aligned curricula. However, they reported that the summer academy was helpful in introducing them to critical components of NGSS and how they can work together to support three-dimensional learning. While the project-based model described in this study is not the only model of an effective approach to NGSS PD, study results suggest that it is certainly one such model that can be utilized and adapted by others.

There was a diversity of aspects of the summer institute that teachers in this study found most helpful. In general, teachers appeared to benefit from the modeling of project-based approaches revolving around driving questions, and engineering design challenges featuring real-world problem solving. One teacher reported a sense of liberation in perceiving the engineering challenge as nearly impossible at first, but discovering that “it was not that hard” upon completion; and she wanted to share that sense of empowerment with her students. Several teachers discussed the benefit of unpacking and mapping the standards to specific units and lessons. Perhaps most of all, teachers were appreciative of the time and opportunity to write an NGSS-aligned curricular unit in collaboration with their peers. Daily activities during the academy and subsequent PLCs provided a forum for teachers who taught the same grade and content area to work together in curricular development. Teacher participants found that the common planning and thinking space provided in both the summer academy workshops and PLCs helped them to flesh out ways to align existing lessons, and receive constructive feedback to guide their lesson planning. We believed that the highly supportive science coordinator had a very positive influence on teachers in this project. Many teachers stated that they are not sure what they would have done for NGSS preparation without the opportunity to participate in the PD.

No teachers identified any part of the PD that was not beneficial. It is possible that this finding was influenced by some measure of response bias since the interviewer was a part of the university team providing the PD. However, there were few parts of the PD that were not positively identified as beneficial.

Participants did identify several elements that would have been helpful for the summer institute to include. This included more model lesson plans and assessments. Because NGSS is still so new, such models are still scarce. However, we attempted to address this need by sharing models of curricula and assessments, and other resources online and during the PLCs. One teacher shared that participating in engineering design activities “as if a student” would be even more helpful if followed by how to facilitate them through teaching.

### Implications for Teacher Professional Development and Educational Practice

We noted some other issues in the process of implementing our PD intervention with implications for practice. In our implementation, the instructional team was committed to providing ample time and opportunity for teachers to write aligned curricula in order to reach the goals of the workshop. Unfortunately, honoring this priority meant sacrificing a deeper treatment of PBL conceptualization than the PD facilitators would have liked. Related to the importance of dosage, there were indications that some teachers may have benefited from this. It is important to reach a strategic balance between the presentation of pedagogical and conceptual models, and opportunities for teachers to collaboratively develop curricula and practice instruction. It also remains important to “walk the talk” in truly *modeling* a project-based approach with respect to how the PD is implemented. We believe that doing so in our project enhanced teacher satisfaction and perceptions of PD benefit.

From the perspective of educational psychology, we also observed that our PD model emphasized some learner-centered psychological principles more than others (McCombs, 1993, 1997; American Psychological Association [APA], 1997). Among the merits of project-based approaches are their tendency to be motivating and collaborative, which address some of the social and emotional influences on learning. Future adaptations of this model, however, could provide more instruction on strategies to support self-regulated learning and metacognition (e.g., Zimmerman, 1990, 2002), or to recognize the influence of development, individual differences, and diversity on learning.

## Conclusion

In this study, we presented a project-based model of teacher PD on NGSS alignment, and evaluated the impact of the PD on teachers’ development through interviews and an analysis of teachers’ lesson plans. Even though most teachers who participated in the PD obtained a basic understanding and conceptualization of NGSS and PBL, and made significant pedagogical and curricular shifts to align with NGSS, their application of this understanding in their curriculum writing varied. This is of potential concern since poorly written and executed curricula can negatively affect students’ motivation, efficacy, and achievement. Overall, however, findings attest to the value of the PD model, as well as a variety of ways to deepen each teacher’s PD experience toward meeting NGSS goals. Future PD efforts should strive to decrease the gap between teacher’s understanding of the core elements of NGSS and their ability to use this understanding to write NGSS-aligned curricula. In addition, future studies should also evaluate teacher’s implementation of their curricula in their daily instruction. Finally, future studies should create more instruments and measures of student understandings of the core components of NGSS to better gauge the effectiveness of project-based and NGSS-aligned curricula and instruction. If these recommendations are heeded, the present study can inform much needed efforts to help teachers align curricula and instruction to NGSS through teacher PD.

## Ethics Statement

This study was carried out in accordance with the recommendations of The Rutgers University Institutional Review Board (IRB) with written informed consent from all subjects. All subjects gave written informed consent in accordance with the Declaration of Helsinki. The protocol was approved by the Rutgers University Institutional Review Board (IRB).

## Author Contributions

DS was Principal Investigator, and led the conception and design of the study, literature review, analyses, interpretation, and writing and revising of the manuscript. SS contributed to the PD program, led data collection, conducted all interviews, contributed to analyses, and contributed to the writing and revising of the manuscript. DB contributed to the analyses, and to the writing and revising of the manuscript. DS co-facilitated the summer institute, and contributed to the writing and revising of the manuscript.

## Conflict of Interest Statement

The authors declare that the research was conducted in the absence of any commercial or financial relationships that could be construed as a potential conflict of interest.

## Footnotes

1 http://www.bie.org/

2 www.ecomodeler.org
