# Supplementary material for: Teacher Perceptions of Their Curricular and Pedagogical Shifts: Outcomes of a Project-Based Model of Teacher Professional Development in the Next Generation Science Standards
Source: Front Psychol. 2017 Jun 16;8:989. doi: 10.3389/fpsyg.2017.00989 (PMC5472689; doi:10.3389/fpsyg.2017.00989)
Supplement: Supplementary file 1 [file Table_1.PDF]

Appendix A. NGSS and PBL Lesson Plan Rubric

Curriculum Elements	Minimal Demonstration of Understanding and Application	Partial or Inconsistent Demonstration of Understanding and Application	Clear Demonstration of Firm Understanding and Application
**1. Key Knowledge, Understanding & Success (PBL).**	Student learning goals are not clear and specific; the project is not focused on standards. The project does not explicitly target, assess, or scaffold the development of success skills.	The project is focused on standards-derived knowledge and understanding, but it may target too few, too many, or less important goals. Success skills are targeted, but there may be too many to be adequately taught and assessed.	The project is focused on teaching students specific and important knowledge, understanding, and skills derived from standards and central to academic subject areas. Important success skills are explicitly targeted to be taught and assessed, including critical thinking/problem solving, collaboration, and self-management.
**2. Challenging Problem or Question (PBL).**	The project is not focused on a central problem or question (it may be more like a unit with several tasks); or the problem or question is too easily solved or answered to justify a project. The central problem or question is not framed by a driving question for the project, or it is seriously flawed. For example, it has a single or simple answer; it is not engaging to students (it sounds too complex or	The project is focused on a central problem or question, but the level of challenge might be inappropriate for the intended students. The driving question relates to the project but does not capture its central problem or question (it may be more like a theme). The driving question meets some of the criteria for an effective driving question in the right column, but lacks others.	The project is focused on a central problem or question at the appropriate level of challenge. The central problem or question is framed by a driving question for the project, which is: open-ended; it will allow students to develop more than one reasonable answer; understandable and inspiring to students; and aligned with learning goals. To answer it, students will need to gain the intended knowledge, understanding, and skills.

	"academic," like it came from a textbook); or it appeals only to a teacher.		
**<u>3. Sensemaking (EQuIP):</u> Making sense of phenomena and/or designing solutions to a problem drive student learning.**	i. Student questions and prior experiences related to the phenomenon or problem *rarely* motivate sense-making and/or problem solving.<br><br>ii. The focus of the lesson *does not effectively support* students in making sense of phenomena and/or designing solutions to problems.	i. Student questions and prior experiences related to the phenomenon or problem *sometimes but inconsistently* motivate sense-making and/or problem solving.<br><br>ii. The focus of the lesson *somewhat effectively supports* students in making sense of phenomena and/or designing solutions to problems.	i. Student questions and prior experiences related to the phenomenon or problem *consistently* motivate sense-making and/or problem solving.<br><br>ii. The focus of the lesson *effectively supports* students in making sense of phenomena and/or designing solutions to problems.
**<u>4. Alignment to NGSS Core Components (EQuIP):</u> Opportunities to development and use of Science and Engineering Practices (SEPs); Disciplinary Core Ideas (DCIs); and Cross-Cutting Concepts (CCCs).**<br><br>Builds understanding of multiple grade-appropriate elements of the science and engineering	i. *Few or limited opportunities* to *develop and use* specific elements of the SEP(s).<br><br>ii. *Few or limited opportunities* to *develop and use* specific elements of the DCI(s).<br><br>iii. *Few or limited opportunities* to *develop and use* specific elements of the CCC(s).	i. *Some but inconsistent opportunities* to *develop and use* specific elements of the SEP(s).<br><br>ii. *Some but inconsistent opportunities* to *develop and use* specific elements of the DCI(s).<br><br>iii. *Some but inconsistent opportunities* to *develop and use* specific elements of the CCC(s).	i. *Provision of a variety of rich opportunities to develop and use* specific elements of the SEP(s).<br><br>ii. *Provision of a variety of rich opportunities to develop and use* specific elements of the DCI(s).<br><br>iii. *Provision of a variety of rich opportunities* to *develop and use* specific elements of the CCC(s).

practices (SEPs), disciplinary core ideas (DCIs), and crosscutting concepts (CCCs) that are deliberately selected to aid student sense-making of phenomena and/or designing of solutions.			
**<u>5. Integrating the Three Dimensions of NGSS:</u> Student sense-making of phenomena and/or designing of solutions requires student performances that integrate elements of the SEPs, CCCs, and DCIs.**	i. Student sense-making of phenomena and/or designing of solutions requiring student performances *do not effectively integrate* elements of the SEPs, CCCs, and DCIs.<br><br>ii. When engineering is a learning focus, it is *not well integrated* with developing disciplinary core ideas from physical, life, and/or earth and space sciences.	i. Student sense-making of phenomena and/or designing of solutions requiring student performances *somewhat effectively or inconsistently integrate* elements of the SEPs, CCCs, and DCIs.<br><br>ii. When engineering is a learning focus, it is *somewhat integrated* with developing disciplinary core ideas from physical, life, and/or earth and space sciences.	i. Student sense-making of phenomena and/or designing of solutions requiring student performances *effectively integrates* elements of the SEPs, CCCs, and DCIs.<br><br>ii. When engineering is a learning focus, it is *well integrated* with developing disciplinary core ideas from physical, life, and/or earth and space sciences
**<u>6. Development of Performance Assessments (PEs, EQuIP):</u> Elicits direct, observable evidence of three-dimensional learning: students are using practices with core ideas and crosscutting**	*PEs do not effectively elicit* direct, observable evidence of three-dimensional learning demonstrating that students are using practices with core ideas and crosscutting concepts to make sense of phenomena	*PEs inconsistently elicit* direct, observable evidence of three-dimensional learning demonstrating that students are using practices with core ideas and crosscutting concepts to make sense of phenomena	*PEs effectively elicit* direct, observable evidence of three-dimensional learning demonstrating that students are using practices with core ideas and crosscutting concepts to make sense of phenomena and/or to design solutions.

**concepts to make sense of phenomena and/or to design solutions.**	and/or to design solutions.	and/or to design solutions.	
**<u>7. Sustained Inquiry (PBL).</u>**	The "project" is more like an activity or "hands-on" task, rather than an extended process of inquiry. There is no process for students to generate questions to guide inquiry.	Inquiry is limited (it may be brief and only occur once or twice in the project; information-gathering is the main task; deeper questions are not asked). Students generate questions, and while some might be addressed, they are not used to guide inquiry and do not affect the path of the project.	Inquiry is sustained over time and is academically rigorous (students pose questions, gather & interpret data, develop and evaluate solutions or build evidence for answers, and ask further questions). Inquiry is driven by student-generated questions throughout the project.
**<u>8. Authenticity (PBL and EQuIP).</u>**	i. The project resembles traditional "schoolwork;" it lacks a real-world context, tasks and tools; and/or it does not make a real impact on the world or speak to students' personal interests.<br><br>ii. The lesson plan does not engage students in authentic and meaningful scenarios that reflect the practice of science and engineering as experienced in the real world.	The project has some authentic features, but they may be limited or feel contrived.	i. The project has an authentic context; involves real-world tasks, tools, and quality standards; makes a real impact on the world; and/or speaks to students' personal concerns, interests, or identities.<br><br>ii. The lesson plan engages students in authentic and meaningful scenarios that reflect the practice of science and engineering as experienced in the real world:<br><br>iii. Students experience phenomena or design problems as directly as possible

			(firsthand or through media representations).<br><br>iv. Students have opportunities to connect their explanation of a phenomenon and/or their design solution to a problem to questions from their own experience.
**9. Monitoring Student Progress (EQuIP), Feedback, and Revision (PBL)**	i. The unit *does not embed formative assessment* processes throughout that evaluate student learning to inform instruction.<br><br>ii. Students get only limited or irregular feedback about their products and work-in-progress, and only from teachers, not peers. Students do not know how or are not required to use feedback to revise and improve their work.	i. The unit *only partially or inconsistently embeds formative assessment* processes throughout that evaluate student learning to inform instruction.<br><br>ii. Students are provided with opportunities to give and receive feedback about the quality of products and work-in-progress, but they may be unstructured or only occur once. Students look at or listen to feedback about the quality of their work, but do not substantially revise and improve it.	i. The unit *embeds formative assessment* processes throughout that evaluate student learning to inform instruction.<br><br>ii. Students are provided with regular, structured opportunities to give and receive feedback about the quality of their products and work-in-progress from peers, teachers, and if appropriate, from others beyond the classroom. Students use feedback about their work to revise and improve it.

Note. The rubric selects and combines essential elements (and criteria for the demonstration and application of understanding) of project-based learning (PBL) from the The Buck Institute for Education's (2017a) Project Design, and the NGSS from the Educators Evaluating the Quality of Instructional Products (EQuIP) Rubric for Science and Mathematics ("EQuIP rubric for lessons & units: Science - version 3.0," 2016), Elements and criteria are reproduced verbatim with minor editing. They were combined from the two sources in a way that addressed project goals.
